# Loss of WT huntingtin impairs ER-to-Golgi transport

**Published:** 2014-12

Huntington’s disease (HD) is an autosomal dominant neurodegenerative disease caused by mutations in the *HTT* gene, which encodes the huntingtin protein. Although the mutant protein is known to form protein aggregates leading to neuronal toxicity, the loss of wild-type (WT) huntingtin functions – which include axonal transport of brain-derived neurotrophic factor (BDNF) – might also play a role in HD. To elucidate this, Folma Buss and colleagues used primary fibroblasts from homozygous mutant *Htt^140Q/140Q^* knock-in mice, *HTT* knockdown human HeLa cells and heterozygous HD patient fibroblasts that express one WT copy and one mutant copy (with a 180Q expansion) of *HTT*. They analysed different steps of protein secretion and found that the complete loss of WT huntingtin in mouse fibroblasts and HeLa cells impaired vesicle transport from the endoplasmic reticulum (ER) to the Golgi complex. Although one copy of WT protein was sufficient to maintain the secretory pathway in human HD fibroblasts, the distances for cargo transport in the secretory pathway are extremely large in neurons; therefore, a slight decrease in the efficiency of delivery might contribute to the reduced BDNF secretion in HD. These results have important implications for therapies that aim to reduce huntingtin levels. **Page 1335**

